# Pleural empyema in a patient with a perinephric abscess and diaphragmatic defect

**DOI:** 10.1002/rcr2.400

**Published:** 2019-01-29

**Authors:** Pei Sze Carmen Tan, Arash Badiei, Deirdre B. Fitzgerald, Yi Jin Kuok, Y. C. Gary Lee

**Affiliations:** ^1^ Department of Respiratory Medicine Sir Charles Gairdner Hospital Nedlands Western Australia Australia; ^2^ Department of Respiratory and Critical Care Medicine Tan Tock Seng Hospital Singapore Singapore; ^3^ Department of Radiology Sir Charles Gairdner Hospital Nedlands Western Australia Australia; ^4^ Centre for Respiratory Health University of Western Australia Perth Western Australia Australia

**Keywords:** Abscess, diaphragmatic defect, empyema, perinephric, pleural

## Abstract

Pleural infection as a complication of ascending urological infection is rare, and the mechanism often unclear. We report a complicated case of pleural infection and perinephric abscess in a patient who presented with a large right‐sided pleural effusion. Pleural fluid culture yielded *Morganella morganii*, an unusual pathogen in pleuro‐pulmonary infections. Her computed tomography (CT) scan of abdomen showed a right perinephric abscess which extended into the pleural cavity. Review of prior CT imaging suggested a pre‐existing diaphragmatic defect, likely representing a congenital Bochdalek foramen, through which the infection ascended. Successful treatment was achieved with systemic antibiotics, and drainage of both the pleural and retroperitoneal collections. Intra‐pleural tissue plasminogen activator/deoxyribonuclease therapy effectively cleared the residual pleural fluid. Spread of intra‐abdominal sepsis through diaphragmatic defects to the pleural cavity represents a potential source of empyema.

## Introduction

Pleural effusions can be associated with a wide range of sub‐diaphragmatic disorders. Ascending urological infections are rare 1; published cases focussed on development of nephro‐pleural fistula following percutaneous nephrolithotomy and causing localized symptoms 2, 3. We report a case of pleural infection secondary to an occult perinephric abscess via a pre‐existing (likely congenital) diaphragmatic defect.

## Case Report

A 68‐year‐old nursing home resident was brought to our emergency department with one week of fever and increasing dyspnoea. Her background history included advanced multiple sclerosis complicated by quadriplegia and mild cognitive impairment, which had rendered her largely bedbound. She was also dually incontinent and required long‐term indwelling urinary catheter. There was a known history of recurrent urinary tract infections, including right‐sided pyelonephritis five years prior. On presentation, she was febrile (39°C), normotensive but tachycardic (130/min) and in respiratory distress. Clinical examination showed signs of a large right pleural effusion but no abdominal tenderness. There were marked peripheral leucocytosis (68 × 10^9^/L) and a raised C‐reactive protein (CRP) level (500 mg/L). Imaging, including computed tomography (CT) thorax, showed extensive peri‐bronchial thickening, right lower lobe consolidation and a complex right pleural effusion with thickened pleura. She was commenced on intravenous piperacillin/tazobactam. A 12F intercostal catheter was urgently inserted and removed 800 mL of turbid, exudative, and acidic (pH 6.6) pleural fluid which grew *Morganella morganii*, an organism usually associated with urological or gastrointestinal infections. Blood and urine cultures were negative. CT of the abdomen showed a right perinephric collection involving psoas muscle and extending into the right thoracic cavity. The right kidney demonstrated atrophic calcification, likely related to previous infections.

**Figure 1:**
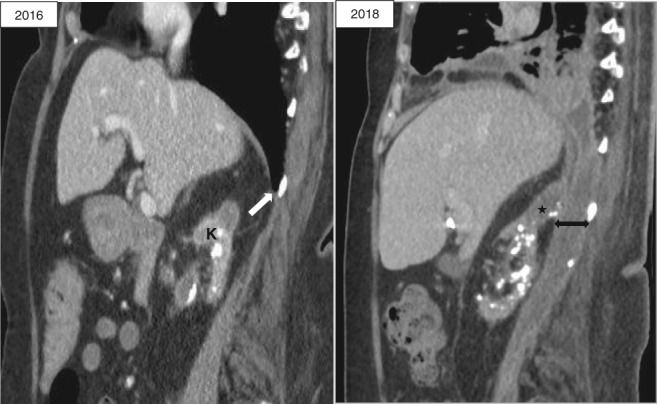
Sagittal computed tomography images of 2016 showing right posterolateral diaphragmatic defect (white arrow) and atrophic kidney (K), allowing transit of perinephric and psoas abscess (star) via the pre‐existing defect (left–right arrow) into pleural cavity in 2018.

**Figure 2:**
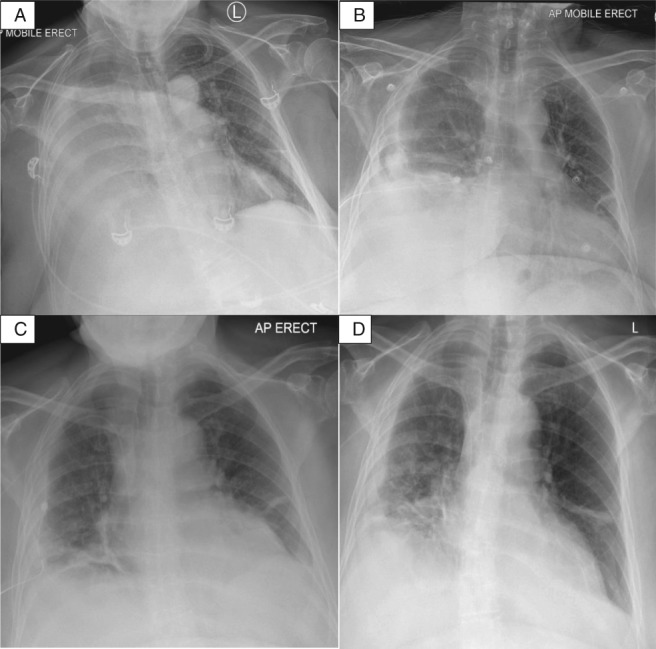
Progression of empyema on chest X‐rays (CXRs) from admission to end of treatment. (A) Large right pleural effusion on admission; (B) after 2 days of chest tube drainage showing a moderate sized residual right loculated effusion; (C) post intrapleural tissue plasminogen activator/deoxyribonuclease therapy; (D) follow‐up CXR 1‐month post‐discharge.

A careful review of her past abdominal CT two years ago revealed a small right‐sided posterolateral (likely Bochdalek type) diaphragmatic defect. This could allow extension of her right retroperitoneal infection into the right thoracic cavity (Fig. 1). She showed initial clinical improvement; by 48 h her leukocyte counts and CRP had decreased to 23 × 10^9^/L and 220 mg/L, respectively. Thereafter she had persistent low‐grade fever and raised inflammatory markers for the following three days. She had a sizeable multi‐loculated residual pleural effusion on ultrasonography and CT. Antibiotic was escalated to meropenem and intrapleural tissue plasminogen activator/deoxyribonuclease (tPA/DNase) (alteplase 5 mg/pulmozyme 5 mg) was initiated twice daily for 3 days with effective clearance of the pleural collection (Fig. 2) and improvement of CRP (to 120 mg/L). Urology review recommended CT‐guided drainage of the retroperitoneal collection as the least invasive option, given her frailty. This yielded 80 mL of pus which also cultured *M. morganii.* She further improved afterwards with sustained defervescence and normalization of inflammatory markers. She was discharged back to residential care with intravenous antibiotics for a further four weeks and had since remained well.

## Discussion

Pleural infection commonly develops when pneumonic processes extend to the pleura. Empyema following abdominal infection has been described but the pathobiological mechanism is poorly understood. Often, it is believed that trans‐diaphragmatic migration of infected abdominal fluid permit bacterial seeding of the pleura. Our patient was unique in that she had a pre‐existing diaphragmatic defect (likely congenital Bochdalek foramen), the proposed route for ascending retroperitoneal infection into the thoracic cavity. *M. morganii*, an organism associated with abdominal infections, was recovered from both the pleural and peritoneal collections, supporting that it was an ascending abdominal‐to‐pleural infection. The organism could have migrated to the pleura directly, for example from a fistula or rupture of the peritoneal abscess, or by infecting the tissues separating the pleura and peritoneum. A diaphragmatic defect would make either pathway easier.

Pleural infection secondary to intra‐abdominal sepsis is an important but easily missed clinical entity that poses a diagnostic challenge, especially where respiratory symptoms predominate, and/or underlying sub‐diaphragmatic pathology is clinically silent. Lumbar back pain and fever were the most common symptoms in patients with renal/perinephric abscesses; conversely dysuria/pyuria were uncommon (<10% of patients) 4.

Intrapleural tPA/DNase therapy is an effective adjunct treatment for pleural infection 5, and has been shown to significantly reduce the need for surgical intervention. To our knowledge, this is the first report of intrapleural tPA/DNase therapy in a patient who had concurrent (and likely communicating) infected pleural and intra‐abdominal collections. In this case, the treatment was effective and caused no pleural or peritoneal complications.

### Disclosure Statement

Appropriate written informed consent was obtained for publication of this case report and accompanying images.
